# Emerging precision neoadjuvant systemic therapy for patients with resectable non-small cell lung cancer: current status and perspectives

**DOI:** 10.1186/s40364-022-00444-7

**Published:** 2023-01-18

**Authors:** Luis A. Godoy, Joy Chen, Weijie Ma, Jag Lally, Kyra A. Toomey, Prabhu Rajappa, Roya Sheridan, Shirish Mahajan, Nicholas Stollenwerk, Chinh T. Phan, Danny Cheng, Robert J. Knebel, Tianhong Li

**Affiliations:** 1 Division of Thoracic Surgery, Department of Surgery, University of California Davis School of Medicine, Sacramento, CA USA; 2 Medical Student, University of California Davis School of Medicine, Sacramento, CA USA; 3 Division of Hematology/Oncology, Department of Internal Medicine, University of California Davis School of Medicine, University of California Davis Comprehensive Cancer Center, Sacramento, CA USA; 4 Medical Service, Hematology and Oncology, Veterans Affairs Northern California Health Care System, Mather, CA USA; 5 Division of Pulmonary, Critical Care, and Sleep Medicine, Department of Internal Medicine, University of California Davis School of Medicine, Sacramento, CA USA; 6 Medical Service, Pulmonology, Veterans Affairs Northern California Health Care System, Mather, CA USA; 7 Department of Radiology, Interventional Radiology, Veterans Affairs Northern California Health Care System, Mather, CA USA

**Keywords:** NSCLC, Resectable, Neoadjuvant, Immune checkpoint inhibitor (ICI), Targeted therapy, Precision oncology, Systemic therapy

## Abstract

Over the past decade, targeted therapy for oncogene-driven NSCLC and immune checkpoint inhibitors for non-oncogene-driven NSCLC, respectively, have greatly improved the survival and quality of life for patients with unresectable NSCLC. Increasingly, these biomarker-guided systemic therapies given before or after surgery have been used in patients with early-stage NSCLC. In March 2022, the US FDA granted the approval of neoadjuvant nivolumab and chemotherapy for patients with stage IB-IIIA NSCLC. Several phase II/III trials are evaluating the clinical efficacy of various neoadjuvant immune checkpoint inhibitor combinations for non-oncogene-driven NSCLC and neoadjuvant molecular targeted therapies for oncogene-driven NSCLC, respectively. However, clinical application of precision neoadjuvant treatment requires a paradigm shift in the biomarker testing and multidisciplinary collaboration at the diagnosis of early-stage NSCLC. In this comprehensive review, we summarize the current diagnosis and treatment landscape, recent advances, new challenges in biomarker testing and endpoint selections, practical considerations for a timely multidisciplinary collaboration at diagnosis, and perspectives in emerging neoadjuvant precision systemic therapy for patients with resectable, early-stage NSCLC. These biomarker-guided neoadjuvant therapies hold the promise to improve surgical and pathological outcomes, reduce systemic recurrences, guide postoperative therapy, and improve cure rates in patients with resectable NSCLC.

## Introduction

**Fig. 1:**
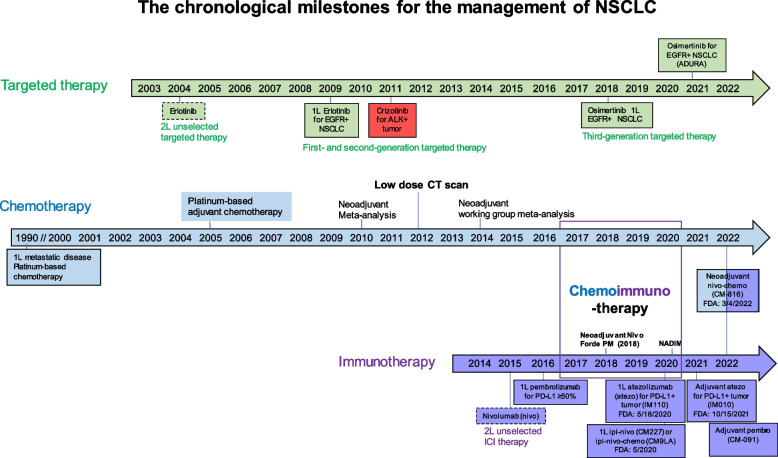
The chronological milestones for the management of NSCLC. Major advances in systemic therapy with chemotherapy, targeted therapy and immunotherapy for NSCLC are illustrated. Beginning in 1990, chemotherapy was shown to be effective for prolonging survival in patients with NSCLC and platinum-based therapy became the cornerstone of treatment. Neoadjuvant or adjuvant chemotherapy modestly increased the overall survival by ~ 5% in patients with stage II-IIIA operable NSCLC. In 2004, targeted therapy erlotinib was approved for *EGFR*-sensitive NSCLC. During this time, researchers began utilizing modern gene sequencing programs to identify genetic markers for targeted therapeutics. The *ALK* gene rearrangement was identified and crizotinib was approved for *ALK*-positive tumors in 2011. In 2015, ICIs were found to improve survival and three immunotherapy agents were approved, including nivolumab, atezolizumab, and pembrolizumab. In 2018, a third-generation EGFR inhibitor osimertinib was approved as first line treatment for patients with metastatic NSCLC. By the end of 2020, adjuvant targeted therapy with osimertinib was approved for *EGFR*-sensitive NSCLC. In 2021, adjuvant immunotherapy with atezolizumab was approved for PD-L1 positive NSCLC. Neoadjuvant nivolumab and platinum-based chemotherapy becomes a standard of care for patients with stage IB-IIIA NSCLC without known *EGFR* or *ALK* genomic alterations in March 2022

**Fig. 2:**
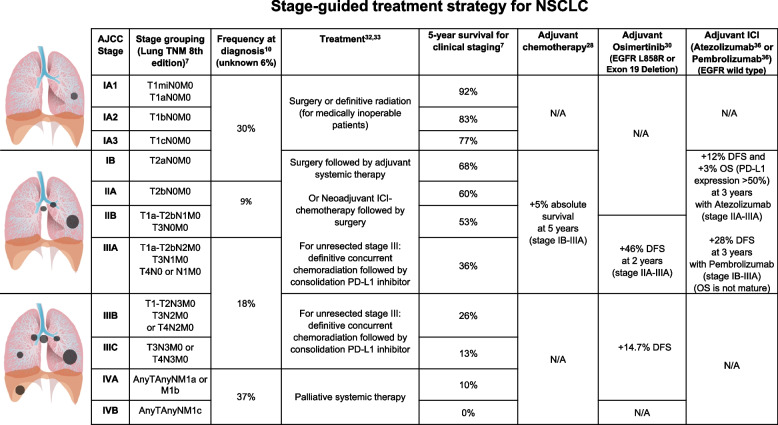
Summary of stage-guided treatment selection for NSCLC. The frequency indicates the stage distribution (%) at diagnosis based on AJCC stage. Survival data is reported for adjuvant chemotherapy (stage IB-IIIA), osimertinib (stage IIA-IIIA), pembrolizumab (stage IB-IIIA), and atezolizumab (stage IIA-IIIA). Increased survival for adjuvant therapy is reported as a percentage increase in comparison to surgery or chemotherapy alone

Lung cancer is the leading cause of cancer-related death in the United States and worldwide [1, 2]. Globally, lung cancer currently contributes to an estimated 2.3 million new cases and 1.8 million per year. In the United States, lung cancer occurs in approximately 236,000 patients with 130,000 deaths in 2020. Lung cancer causes more deaths than breast, prostate, colorectal, and brain cancers combined [1]. The survival rates differ among lung cancer by histological type, stage at diagnosis, and treatment [3, 4]. Non-small cell lung cancer (NSCLC) comprises 80–85% of lung cancer, followed by 10–15% of small cell lung cancer, < 5% of lung carcinoid or other types of lung cancer. NSCLC can be further divided into adenocarcinoma, squamous cell carcinoma, large cell carcinoma, and other rare histological types. The American Joint Committee on Cancer (AJCC) is the most used staging system in clinical settings [5–7]. The 7th and 8th editions of AJCC are currently used in neoadjuvant and adjuvant clinical trials to describe stage distribution, prognosis, and treatment selection. Most patients with lung cancer are diagnosed at the incurable, advanced stage and contribute to the majority of lung cancer deaths. When left untreated, lung cancer progresses rapidly with a mean survival of about 7 months [8]. Improvement for patients with SCLC is limited, with a 5-year survival rate of 7% [9]. Over the past 3 decades, many advances have contributed to decreased lung cancer incidence and improved overall survival for patients with NSCLC [3, 4]. These advances include primary prevention from smoking cessation, early detection of lung cancer in heavy smokers by low-dose chest CT scan, precise clinical staging through the use of positron emission tomography/computed tomography (PET/CT) scan, and pathological mediastinal staging, improved local therapy with surgery or radiation, the use of adjuvant chemotherapy, molecular targeted therapy and immunotherapy for patients with NSCLC, histology-directed systemic chemotherapy, tumor genotyping, increased use of molecularly targeted therapy and cancer immunotherapy [3, 4]. Figure 1 summarizes the chronological milestones for the management of NSCLC. The 5-year relative survival of all lung cancers is 22%[1]. The 5-year survival for patients with stage I NSCLC is 68–92%, stage II 53–60%, stage IIIA 36%, IIIB-IV 0–26% [10]. The stage-based clinical management for NSCLC and the clinical efficacy of adjuvant systemic therapy with chemotherapy, targeted therapy, and ICI therapy, respectively, are summarized in Fig. 2. Recently, several clinical trials showed significantly improved clinical efficacy with promising increased cure rates using adjuvant and neoadjuvant targeted therapy and immune checkpoint inhibitors (ICI) after or with chemotherapy in patients with resectable NSCLC. This emerging precision neoadjuvant systemic therapy platform is an extension of precision medicine in lung cancer. However, clinical implementation of this precision neoadjuvant treatment strategy requires multidisciplinary team coordination, extensive knowledge of tumor biology, and coordination of immune and molecular biomarker testing. In this article, we review these new developments, challenges in biomarker testing and endpoint selections, and practical considerations in neoadjuvant therapy for patients with operable, early-stage NSCLC.

### Precision oncology in patients with non-operable mNSCLC

The landscape for the diagnosis and treatment of patients with non-operable, locoregionally advanced or metastatic NSCLC has evolved significantly in the past two decades [11] enabling the current era of precision lung cancer care. At diagnosis, NSCLC tumors are subjected to the multiplexed molecular and immune biomarker assays, as either companion or complementary diagnostics for targeted therapy or ICI treatment selection according to the National Comprehensive Cancer Network (NCCN) guidelines [12]. First, small molecular tyrosine kinase inhibitors (TKIs) effectively target a growing number of *gain-of-function* molecular targets (i.e., driver oncogenes), such as sensitizing mutations in the EGFR, BRAF V600E, MET exon 14 skip or ERBBR2 (HER2) genes, and gene rearrangements in the ALK, ROS1, RET, MET, and neurotrophic receptor tyrosine kinase (NTRK) genes, regardless of their tumor PD-L1 expression. These targeted therapies have improved the progression-free survival (PFS) and overall survival (OS) with favorable toxicity profiles compared to platinum-containing combination chemotherapy as first-line systemic therapy in 25–30% of non-operable NSCLC patients with variable oncogene driven mutations. Tumor genomic typing of NSCLC is important before starting the ICI therapy, not only because ICIs have no or inferior clinical effect in oncogene driven tumors, but because they have also been associated with increased incidence and severity of interstitial lung disease and other ICI-related toxicities when they are in sequential or concurrent use with EGFR TKIs in patients with metastatic NSCLC [13, 14]. Secondly, in non-operable NSCLC patients with no EGFR or ALK genomic tumor aberrations, first-generation PD-1/PD-L1 inhibitors, either as monotherapy or in combination with platinum-containing combination chemotherapy, has become the standard of care first line treatment. Furthermore, adding anti-CTLA-4 to anti-PD-L1 alone or with chemotherapy confers additional clinical and long-term survival benefit in selected patient populations [15, 16]. Uptake of ICI, which was only approved by the US Food and Drug Administration in 2015, has been rapid. In 2018, approximately 33% of patients with newly diagnosed, metastatic NSCLC received ICI, up from 12% in 2016 [10]. Despite these advances, the overall 5-year survival for SCLC is 7% and NSCLC is 26% overall and for all stages combined [10]. Nevertheless, the 5-year survival for nearly 50% of metastatic NSCLC patients with either oncogene-driven genomic alterations or immune checkpoint biomarkers have exceeded 15–20% in 2019 and > 25% in 2020 [17]. The expression of PD-L1 on the surface of tumor cells and immune cells, detected by immunohistochemistry, is the most used predictive biomarker used to guide treatment decisions with anti-PD-1 or anti-PD-L1 antibodies in patients with NSCLC. In addition, several tumor and blood molecular characteristics have been associated with increased clinical response to PD-1/PD-L1 inhibitors in NSCLC [18, 19]. The clinical implementation of precision oncology in lung cancer requires close collaboration of multidisciplinary team, along with further technology and knowledge advances in understanding tumor biology and biomarker-guided treatment selection at the patient level.

### Perioperative chemotherapy for resectable, early-stage NSCLC

Surgery remains the best treatment modality for curing patients diagnosed with resectable stage I-IIIA NSCLC [17, 20]. The increased use of screening low-dose CT scans in high-risk patients for lung cancer has led to the identification of more early-stage patients who can be cured with local therapy [21]. In the United States, the proportion of lung cancer disease diagnosed at a localized stage increased from 17% during the mid-2000s to 20% in 2013 and 28% in 2018 [22]. However, surgery and general anesthesia can have severe complications, requiring assessments for pulmonary and cardiac function [5]. For those patients with inoperable early-stage NSCLC due to medical limitations in surgery, stereotactic body radiotherapy (SBRT) becomes a standard treatment with local control rates comparable to surgical series [23]. Up to 50% of NSCLC patients can have tumor recurrence after surgery [24], mostly from distant metastasis. Perioperative chemotherapy has been used to decrease tumor recurrence.

The goal of neoadjuvant or induction therapy is to reduce tumor burden prior to definitive therapy. Neoadjuvant cisplatin-based chemotherapy improves both survival and cure (5-year DFS) in unselected participants with stages IB-IIIA lung cancers to a degree at least equal to adjuvant use of the same chemotherapies [25, 26]. A meta-analysis of 15 randomized controlled neoadjuvant trials of 2,385 patients demonstrated that OS was significantly improved with preoperative chemotherapy followed by surgery compared with surgery alone (HR, 0.87; 95% CI, 0.78–0.96; *P* = 0.007) [25]. Preoperative chemotherapy translated into a 5% absolute improvement in OS at 5 years. Recurrence-free survival (RFS) was also improved with neoadjuvant chemotherapy (HR, 0.85; 95% CI, 0.76–0.94; *P* = 0.002). The absolute increase in RFS at 5 years was 6%. Additionally, neoadjuvant chemotherapy decreased distant recurrence rate (HR, 0.69; 95% CI, 0.58–0.82; *P* < 0.0001). The absolute increase in freedom from distant recurrence at 5 years was 10%. Rates of complete pathologic response (pCR) are 5–10% and major pathologic response (MPR) are 11–20% with cisplatin-based chemotherapy [25, 26]. The time to locoregional recurrence was not significantly improved with preoperative therapy, suggesting its OS benefit is mainly secondary to decreased rates of distant recurrence. As the neoadjuvant and adjuvant chemotherapy have similar modest 5-year survival benefit and low pCR (i.e., 5–10%) neoadjuvant chemotherapy has not been routinely used for the majority of early-stage NSCLC who are good surgical candidates. There is an unmet need to develop new systemic therapies to further improve the clinical outcomes of patients with resected NSCLC. Recently, perioperative targeted and immune checkpoint inhibitor therapies have been shown to significantly increase the pathological tumor response and recurrence-free survival without significant risk of tumor progression in patients with resectable NSCLC.

### Recent advances of precision adjuvant systemic therapy

Recently, adjuvant targeted therapy with osimertinib for EGFR-sensitive NSCLC (ADAURA) [27] and immunotherapy with atezolizumab for PD-L1-positive NSCLC (IMpower010) [28] have also been shown to increase the survival, and become standard of care for patients with resected stage I-III NSCLC [29, 30]. American Society of Clinical Oncology (ASCO) and NCCN have incorporated these data into their clinical practice guidelines for adjuvant systemic therapy for patients with resected stage I-III NSCLC [29, 30]. For patients with Stage IB (3 < T ≤ 4 cm, N0M0) and sensitizing *EGFR* (Ex19del or L858R) mutations, 3-year adjuvant osimertinib is recommended without chemotherapy. For patients with stages IIA, IIB, and IIIA, adjuvant cisplatin-based chemotherapy is recommended for all fitted patients. Adjuvant osimertinib is recommended after chemotherapy for patients with tumors with sensitizing EGFR mutations, regardless of the PD-L1 status. Adjuvant atezolizumab is recommended for all patients with PD-L1 ≥ 1% after cisplatin-based chemotherapy except for patients with sensitizing EGFR mutations. The clinical uptake of these practice guidelines is limited.

However, there are still many unanswered questions for optimizing adjuvant systemic therapy for resectable NSCLC. First, several molecular risk stratification assays and novel cancer biomarkers, independent of clinical characteristics, are available to determine the risk of cancer recurrence and select high-risk patients for adjuvant chemotherapy [31–33]. Their roles in predicting clinical response to adjuvant immunotherapy needs to be studied. Second, given that neoadjuvant nivolumab and chemotherapy received FDA approval for in March 2022, it remains to be seen if adjuvant or neoadjuvant chemoimmunotherapy provides improved survival or even cure in patients with resectable NSCLC. Third, the role of PD-L1 IHC in selecting NSCLC patients for adjuvant ICI therapy remains unclear. In an interim analysis of a phase III trial (PEARLS/KEYNOTE-091 study), one-year pembrolizumab treatment significant improved median DFS over placebo (53.6 versus 42.0 months; HR, 0.76; 95% CI, 0.63–0.91; *P* = 0.0014) in patients with stage IB-IIIA NSCLC as an adjuvant treatment following surgical resection (lobectomy or pneumonectomy), regardless of PD-L1 expression [34, 35]. In the TPS ≥ 50% subgroup, median DFS had yet to be reached in either treatment arm. At the time of this analysis, DFS events were observed in 32% of the pembrolizumab group and 38% of the placebo group. The KEYNOTE-091 study did not mandate adjuvant chemotherapy, and approximately 14% of patients did not receive adjuvant chemotherapy. Patients who received adjuvant chemotherapy and those with squamous histology had numerically better DFS with placebo. Thus, chemotherapy seems to be essential to the effectiveness of ICI. Ongoing phase III Chemo-IO (ACCIO) study aims to determine the role of adjuvant pembrolizumab with or after chemotherapy in resected NSCLC [36].

### The immunosuppressive effect of surgery during peri-operative period

Surgery and anesthesia induce various metabolic and endocrine responses, which result in a generalized state of immunosuppression in the immediate post-operative period (days to weeks). Since initial reports of the prometastatic effects of surgery in early 1900s [37], numerous studies have been conducted to understand the mechanisms of surgery-induced immunosuppression in the development of cancer metastasis and poor clinical outcomes [38–41]. Surgery-induced prometastatic effect is linked to tumor cell dissemination to nearby blood vessels, organs, and lymphatics [42]. In addition, there are local and systematic release of growth factors (e.g. VEGF) [43], as well as impairment of innate and adaptive immunity [44–46]. Thus, the frequency of metastatic spread correlates with the degree of surgical trauma during the operation [39]. Adjuvant platinum-based systemic chemotherapy has been shown to decrease local and distant recurrence, presumably by eliminating factors that contribute to perioperative micrometastasis.

Activation of the sympathetic nervous system during surgery also has a profound deleterious effect on the immune system. Central neuroendocrine hormonal responses occur in response to trauma. Immune organs or lymphoid organs are innervated with nerve fibers that are directly stimulated by the sympathetic nervous system. When stimulated, there is a release of catecholamines which have an immunosuppressive effect [47]. In addition, stress activates the hypothalamic–pituitary–adrenal axis (HPA), releasing adrenocorticotropic hormone (ACTH) and glucocorticoids, mediating the effect of surgery on the immune system [48]. These effects may persist for several days following surgery and is proportional to the severity of the surgical stress. Additional factors, such as intraoperative blood loss, hypothermia and postoperative sepsis, also contribute to postoperative immune suppression [49]. Compared to conventional open surgery, minimally invasive techniques decrease the degree of surgical trauma and preserves lymphocyte subpopulations, neutrophil function, and cell mediated immunity. They also contribute to reduced post-operative complication rate and shorter hospital stays [38, 50].

### Preclinical rationales of neoadjuvant immunotherapy

As lung cancer and surgical intervention can be prometastatic and immunosuppressive, neoadjuvant immunotherapy can attack micromestasis early and mitigate its immunosuppressive effects to improve surgical and survival outcomes [41, 51]. ICIs are designed to activate exhausted tumor-reactive T cells, which are responsible for killing tumor cells. Current data suggest that the presence of high membranous PD-L1 IHC staining on tumor cells and the presence of intratumoral PD-1 expressing tumor infiltrating lymphocytes (TILs) in the tumor microenvironment (TME) are favourable prognostic factors and the best predictive factors of clinical response to ICIs [19]. A T-cell inflamed gene expression profile (GEP) in addition to PD-L1 IHC has improved the prediction of favourable clinical response to ICIs [19]. ICIs could increase the number of absolute lymphocyte counts (ALCs), restore the function in exhausted CD8 + T cells and induce phenotypical and functional changes of effector immune cells [52]. Recent studies showed that early stage and late-stage NSCLC have distinct tumor biology [53]. Neoadjuvant or preoperative injection of anti-PD-1 inhibitors might be advantageous to activate tumor infiltrating T-cells prior to surgery and to avoid the reduction of PD-1 expression on immune cells in the postoperative period.

### Emerging data of neoadjuvant ICI and chemotherapy for non-oncogene-driven NSCLC

**Fig. 3:**
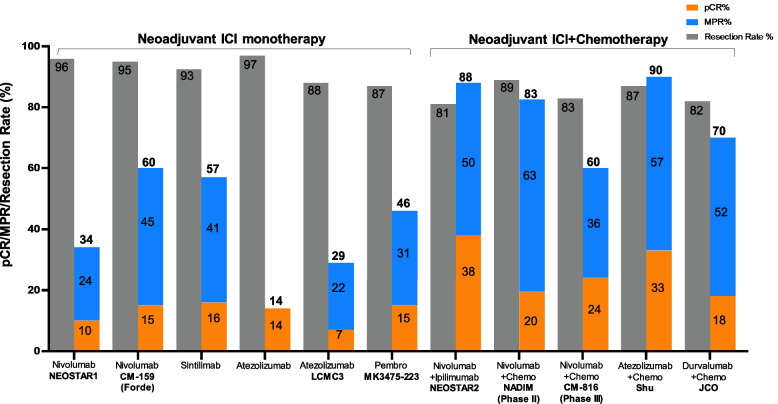
Surgical and pathological outcomes of neoadjuvant ICI alone or with chemotherapy studies. Data is stratified by complete response (pCR) rate (%), major pathologic response (MPR) rate (%), and the resection rate (%)

Table 1 summarized the reported neoadjuvant trials in patients with early-stage NSCLC. Over 80% of NSCLC patients were able to have complete tumor resection. Unlike the trials conducted in patients with non-operable, locally advanced NSCLC, ICI monotherapy seems to have less effect compared to the ICI and chemotherapy combination as evaluated by pCR and MPR (Fig. 3). Taking nivolumab as an example, the clinical efficacy is increased when chemotherapy was added. In the first pilot study [54], neoadjuvant nivolumab monotherapy (given at a dose of 3 mg/kilogram of body weight every 2 weeks for 2 doses) was well tolerated without any new adverse events (perioperative adverse events of grade 3 or 4 for 90 days after the administration of the last nivolumab dose) and did not delay surgery (as defined by no more than 37 days). Of the 21 patients who had evaluable radiographic results, 2 patients (10%) had a partial response, 18 (86%) had stable disease, and 1 (5%) had disease progression. Of the 21 tumors that were removed, 20 (95%) were completely resected and pathological down-staging from the pretreatment clinical stage occurred in 8 patients (40%). MPR was observed in 9 (45%) tumors. The tumor mutational burden but not PD-L1 expression by IHC was predictive of the pathological response to PD-1 blockade. Treatment induced expansion of mutation-associated, neoantigen-specific T-cell clones in peripheral blood. The number of T-cell clones that were found in both the tumor and peripheral blood increased systemically after PD-1 blockade in 8 of 9 patients who were evaluated. Mutation-associated, neoantigen-specific T-cell clones from a primary tumor with a pCR on pathological assessment rapidly expanded in peripheral blood at 2 to 4 weeks after treatment; some of these clones were not detected before the administration of nivolumab [54]. At a median of 12 months of postoperative follow-up (range, 0.8 to 19.7), 16 of 20 patients (80%) who had undergone surgical resection were alive and recurrence-free. The recurrence-free survival rate at 18 months was 73% (95% CI, 53 to 100).

**Table 1:** Reported Clinical Activity of Neoadjuvant Trials in Patients with NSCLC

**Drug class**	**Drug name** (treatment duration)	**Name** (clinical trial identifier)	**Phase**	**No. of patients**	**Study subject**	**Biomarker(s)**	**Surgical Feasibility**	**TMN Downstage**	**Clinical Efficacy**				**Author** (year) [reference]
									pCR (%)	MPR (%)	PFS/EFS (mo)	OS (mo)	
TKI monotherapy	Erlotinib (56 days)	NCT01217619	II	19	stage IIIA-N2 EGFR mutation-positive NSCLC stage IIIA-N2 EGFR mutation-positive NSCLC Stage IIIA-N2 *EGFR* mutation-positive NSCLC	EGFR mutation exon 19 or 21	74% of patients had resection	21.2%	42.1%		11.2	51.6	Xiong L (2019) [55]
	Erlotinib (42 days)	NCT01407822 (EMERGING)	II	72	Stage IIIA-N2 *EGFR* mutation-positive NSCLC	EGFR mutation exon 19 or 21	84% of patients in the erlotinib arm (64.9% lobectomy; 13.5% bilobectomy; 5.4% pneumonectomy) versus. 68% of patients in the chemotherapy group (54.3% lobectomy; 14.3% bilobectomy; 0% pneumonectomy)	10.8%	54.1%		21.5	45.8	Zhong W (2019) [56]
	Crizotinib (median 30 days; range 28–120)	NA	II	11	NSCLC with N2 disease, no bulky lymph nodes, *ALK* positive	ALK ROS1	91% had R0 resection (10 lobectomy and 1 pneumonectomy)	27.3%	No pCR but 90% PR		NA	NA	Zhang C (2019) [57]
Antiangiogenetic inhibitor with chemotherapy	Nintedanib + Cisplatin + Docetaxel	NCT02225405	I/II	21	Stage IB–IIIA	PDGFR FGFR VEGFR FLT3	90% patients had resection (18 R0 and 1 R1 resection). Of the surgeries, 84% lobectomy, 5% lobectomy and wedge, 11% pneumonectomy		30%		66% at 12 mo	91% at 12 mo	Cascone T (2020) [58]
	Bevacizumab (56 days) + Cisplatin + Docetaxel (77 days)	NCT00130780	II	50	Stage IB-IIIA	VEGF	82% of patients had resection. Of the surgeries, 9% pneumonectomy, 6% bilobectomy, 4% wedge resection, 81% R0 resection	38%	45%		54	3-year OS was 62%	Chaft J (2013) [59]
ICI with chemotherapy	Cycle 1: Paclitaxel + Cisplatin or Carboplatin Cycles 2 and 3 Paclitaxel + Cisplatin or Carboplatin + Ipilimumab (no duration stated)	NCT01820754 (TOP1201 IPI)	II	55	Stage IB, IIA, IIB, or IIIA	CTLA-4	100% of patients had resection in both groups Ipilimumab group: 100% R0 resections versus. chemotherapy group: 98% R0 resections and 2% R1 resections	38%	15%				Yang C (2018) [60]
	Nivolumab + Paclitaxel + Carboplatin (63 days) Followed by Nivolumab monotherapy for 1 year	NCT03081689	II	46	Stage IIIA	PD-L1	89% of patients had resection				77.1% at 24 mo		Provencio M (2020) [61]
	Nivolumab + platinum doublet versus platinum doublet	NCT02998528 (CheckMate 816)	III	358	Stage IB-IIIA	PD-L1	83% of patients in the Nivolumab group (77% lobectomy and 17% pneumonectomy) versus. 78% of patients in the chemotherapy group (61% lobectomy and 25% pneumonectomy)						Spicer J (2021) [62]
	Nivolumab + chemotherapy	NCT03838159 (NADIM II)	II	90	Stage IIIA	PD-L1	92.5% of patients treated with NIVO + chemo had R0 resection versus 65% of patients with chemo	69.8%	36.2%	52%	89.3% at 12 mo. and 66.6% at 24 mo	98.2% at 12 mo. and 84.7% at 24 mo	Provencio M (2022) [63]
ICI monotherapy	Nivolumab (28 days)	NCT02259621	II	21	Stage I, II, or IIIA	PD-L1	95% of patients had complete tumor resection	40%		45%			Forde PM (2018) [64]
	Sintilimab	ChiCTR-OIC-17013726	Ib	40	Stage IA-IIIB	PD-L1	92.5% of patients had resection (60% lobectomies and 32.5% pneumonectomies)			40.5%			Gao S (2020) [65]
	Nivolumab or Nivolumab + Ipilimumab (29 days)	NCT03158129 (NEOSTAR)	II	44	Stage I-IIIA	PD-L1 CTLA-4	95% of patients resected in Nivolumab group; 81% in Nivolumab + Ipilimumab group		Nivolumab: 10% Nivolumab + ipilimumab: 38%	Nivolumab: 22% Nivolumab + ipilimumab: 38%			Cascone T (2021) [66]
	Nivolumab + Ipilimumab (42 days?)	NCT02259621	Ib/II	15	Stage IB (≥ 4 cm)-IIIA	PD-L1 CTLA-4	67% patients had definitive resection		33%		50% are disease free		Reuss JE (2020) [67]
	Atezolizumab (22 days)	NCT02927301 (LCMC3)	II	101	Stage IB-IIIB	PD-L1	89% of patients had resection			18%			Kwiatkowski DJ (2019) [68]
	Pembrolizumab (42 days)	NCT02938624	I	10	Stage I-II	PD-L1				40%			Bar J (2019) [69]
	Atezolizumab (84 days)	NCT02716038	II	30	Stage IB-IIIA	PD-L1	87% of patients had R0 surgical resection. Of the surgical resections, 73% lobectomy, 15% bilobectomy, 12% pneumonectomy)			57%			Shu C (2020) [70]

*Abbreviations: TMN* tumor, node, and metastasis, *pCR* pathological complete response, *MPR* major pathological response, *DFS* disease free survival, *EFS* event free survival

NEOSTAR was the first randomized phase II trial comparing nivolumab monotherapy and nivolumab and ipilimumab combination for 3 cycles before surgery [71]. Forty-four patients were enrolled in each arm. Compared with nivolumab monotherapy, nivolumab plus ipilimumab resulted in higher pCR rates (10% versus 38%), less viable tumor (median 50% versus 9%), higher MPR (50% versus 24%), and greater frequencies of effector, tissue-resident memory T cells [66]. High percentages of baseline tumor cells with expression of PD-L1 was positively correlated with radiographic responses and pathologic tumor responses at the time of surgery.

Neoadjuvant chemotherapy and nivolumab in resectable non-small-cell lung cancer (NADIM) is a single-arm, phase 2 trial that evaluated the safety and efficacy of neoadjuvant nivolumab and chemotherapy combination in resectable stage IIIA NSCLC [61]. Of the 51 patients screened, 46 patients were enrolled and treated with neoadjuvant paclitaxel (200 mg/m2 once a day) and carboplatin (area under curve 6) plus nivolumab (360 mg) once on day 1 of each 21-day cycle, for three cycles, followed by adjuvant nivolumab monotherapy for 1 year (240 mg once every 2 weeks for 4 months, followed by 480 mg once every 4 weeks for 8 months). The ORR according to radiological criteria was 70% (21 of 30 patients) and included 3 complete responses (10%) and 18 partial responses (60%). Among the 41 patients who underwent surgery, 34 (83%) achieved MPR. At 24 months, PFS was 77.1% (95% CI, 59.9–87.7). 43 (93%) of 46 patients had treatment-related adverse events during neoadjuvant treatment, and 14 (30%) had treatment-related adverse events of grade 3 or worse; however, none of the adverse events were associated with surgery delays or deaths [61]. Updated 3-year OS was 81.9% (95% CI, 66.8–90.6) in the intention-to-treat population, rising to 91.0% (95% CI, 74.2–97.0) in the per-protocol population [72]. Neither TMB nor PD-L1 IHC staining was predictive of survival. Conversely, low pretreatment levels of ctDNA were significantly associated with improved PFS and OS (HR, 0.20; 95% CI, 0.06–0.63, and HR, 0.07; 95% CI, 0.01–0.39, respectively). Clinical responses according to RECIST v1.1 criteria did not predict survival outcomes. However, undetectable ctDNA levels after neoadjuvant treatment were significantly associated with PFS and OS (HR, 0.26; 95% CI, 0.07–0.93, and HR, 0.04; 95% CI, 0.00–0.55, respectively). The C-index to predict OS for ctDNA levels after neoadjuvant treatment (0.82) was superior to that of RECIST criteria (0.72). Thus, ctDNA levels were significantly associated with OS and outperformed radiologic assessments in survival prediction.

CheckMate 816 trial is the randomized phase III study evaluating neoadjuvant chemotherapy with nivolumab plus platinum-doublet chemotherapy in 358 newly diagnosed patients with resectable stage IB to IIIA NSCLC [73]. Patients with known sensitizing EGFR or ALK mutations were excluded. Patients were stratified by cancer stage, PD-L1 status, and gender. Patients were randomly assigned 2:1 to receive nivolumab at 360 mg every 3 weeks plus chemotherapy every 3 weeks (three cycles) versus the same chemotherapy schedule. Then, patients underwent radiologic staging and surgery within 6 weeks of neoadjuvant therapy. They had the option of adjuvant therapy with or without radiation therapy. An exploratory arm of nivolumab plus ipilimumab was closed early. The primary endpoint was pCR by blinded independent review, defined as no residual viable tumor in resected primary tumor and lymph nodes after surgery. Ninety-eight% of all patients enrolled in the trial received neoadjuvant therapy; 94% of the nivolumab-containing arm and 84% of the chemotherapy arm completed treatment, and 83% and 75%, respectively, completed surgery. Lung-sparing surgery (lobectomy) was performed in 77% versus 61%, respectively. Baseline characteristics were well balanced between the two treatment arms. About two-thirds had stage IIIA disease, and participants were evenly split between squamous and nonsquamous histologies. They found that neoadjuvant chemotherapy with nivolumab plus platinum-doublet chemotherapy significantly improved pCR rates compared with chemotherapy alone in patients with resectable stage IB to IIIA NSCLC (24% versus 2.2%, *P* < 0.0001). The magnitude of pathologic complete response (pCR) benefit with nivolumab was similar in patients with stage IB and IIIA disease, in squamous and nonsquamous histologies, regardless of PD-L1 status and TMB. The MPR rate in patients who went on to surgery was 46.8% in the nivolumab-containing arm versus 12.7% for chemotherapy alone. The radiographic objective response rate (ORR) based on scans was 54% with nivolumab plus chemotherapy versus 37% with chemotherapy alone. At a minimum follow-up of 21 months, the co-primary endpoint of median event free survival (EFS) was 31.6 months for the nivolumab plus chemotherapy group and 20.8 months for the chemotherapy alone group. This corresponded to a significant reduction in the risk for disease progression, recurrence, or death of 37% in favor of nivolumab (HR, 0.63;95%CI, 0.45–0.87; *P* = 0.0052). The addition of nivolumab did not appear to increase all-cause adverse events. Adverse events were relatively similar between the two treatment arms. The rate of grade 3 and 4 adverse events was 34% in the nivolumab-plus-chemotherapy arm versus 37% in the chemotherapy-alone arm. Grade 5 surgery-related adverse events were reported in two patients in the chemotherapy arm unrelated to the study drugs. No treatment-related deaths were reported. On March 4, 2022, the US FDA approved nivolumab with platinum-doublet chemotherapy for adult patients with resectable NSCLC in the neoadjuvant setting.

Although CM-816 trial did not mandate adjuvant systemic therapy after surgery, several ongoing phase III trials have included adjuvant ICI therapy. Table 2 and Table 3 summarize ongoing neoadjuvant clinical trials containing either ICI alone or with chemotherapy for NSCLC patients registered at clinicaltrials.gov., respectively. In addition of chemotherapy, many studies are evaluating ICI with either other immunotherapy, chemotherapy, or targeted therapy for patients with early-stage NSCLC as summarized in Table 4. Currently, there are no sufficient data to support the use of radiation as neoadjuvant therapy for resectable NSCLC. In a phase III trial of induction chemoradiation in patients with resectable, stage IIIA/N2 NSCLC, radiation did not add any benefit to indication chemotherapy followed by surgery [74]. In a small, randomized, phase II study (N = 30 each arm), the addition of 2 cycles of neoadjuvant durvalumab to stereotactic body radiotherapy (SBRT) to primary tumor was well tolerated and associated with a high MPR rate compared to SBRT alone (53.3% vs 6.7%; HR, 16.0; 95% CI, 3∙2–79∙6; *p* < 0.0001) in patients with resectable stage I-IIIA NSCLC [75]. Ongoing studies are evaluating the role of radiation in neoadjuvant ICI therapy in resectable NSCLC (Table 5).

**Table 2:** Ongoing Neoadjuvant Clinical Trials Containing ICI Monotherapy for Patients with NSCLC

**ClinicalTrials.Gov Identifier**	**Biomarker(s)**	**Regimen**	**Phase**	**Sample Size**	**Stage**	**Primary Endpoint**	**Estimated or Actual Study Period [reference]**
NCT04941417	PD-L1 expression	PD-(L)1 inhibitor with chemotherapy	II	60	I (T ≥ 4 cm), IIA, IIB, IIIA, IIIB	MPR	November 24, 2020—June 30, 2023
NCT03197467 (NEOMUN)	PD-L1 expression	Pembrolizumab	II	30	II-IIIA	Adverse events, number of patients treated, clinical response, pathologic response	June 18, 2018—October 2023
NCT03053856	PD-L1 expression	Pembrolizumab	II	37	IIIA (N2)	DFS	February 14, 2017—August 13, 2021
NCT04245514	PD-L1 expression	Durvalumab	II	90	T1-4 > 7 N2 M0	EFS	July 15, 2020—March 2025
NCT02994576	PD-L1 expression	Atezolizumab	II	60	IA (≥ 2 cm)-IIIA	Rate of patients without major toxicities or morbidities	December 20, 2016—December 2022
NCT04047186	PD-L1 expression	Nivolumab	II	50	At least one lesion is diagnosed as NSCLC from biopsy pathology. not less than two GGNs on chest CT	PRR	October 1, 2019—December 31, 2024
NCT04062708 (CHIO3)	PD-L1 expression	Durvalumab	II	55	IIIA, IIIB	N2 nodal clearance	March 10, 2021—September 2024
NCT04371796	PD-L1 expression	Sintilimab	II	20	II-IIIA	MPR	May 10, 2020—December 30, 2021

*Abbreviations: CT* computerized tomography, *pCR* pathological complete response, *MPR* major pathological response, *DFS* disease free survival, *EFS* event free survival, *GGNs* ground-glass nodules, *PRR* pathological response rate

**Table 3:** Ongoing Neoadjuvant Clinical Trials Containing ICI with Chemotherapy for Patients with NSCLC

**ClinicalTrials.Gov Identifier**	**Biomarker(s)**	**Regimen**	**Phase**	**Sample Size**	**Stage**	**Primary Endpoint**	**Estimated or Actual Study Period [reference]**
NCT02572843	PD-L1 expression	Durvalumab + Cisplatin/Docetaxel +	II	68	IIIA (N2)	EFS	June 16, 2016—December 2021 [76]
NCT03425643 (KEYNOTE-671)	PD-L1 expression	Pembrolizumab + platinum doublet versus platinum doublet	III	786	IIB, IIIA	EFS, OS	April 24, 2018—June 29, 2026
NCT03456063 (IMpower-030)	PD-L1 expression	Atezolizumab + platinum doublet versus platinum doublet	III	302	II-IIIA, select IIIB (T3N2)	MPR	April 24, 2018—January 20, 2025
NCT02818920 (TOP1501)	PD-L1 expression	Pembrolizumab (neoadjuvant + adjuvant)	II	32	IB-IIIA	Surgical feasibility	January 2017 – March 2026
NCT04304248 (NeoTAP01)	PD-L1 expression	Toripalimab + albumin-bound Paclitaxel + Carboplatin	II	30	IIIA and IIIB with T3N2 disease	MPR	August 1, 2019—July 30, 2026
NCT04422392	PD-L1 expression	Carboplatin, Pemetrexed or Nab-paclitaxel, PD-1 antibody	II	107	IIIA- N2	PFS	July 13, 2020—June 30, 2025
NCT03480230	PD-L1 expression	Compound 121,564 + chemotherapy depending on tumor histology	II	60	IB (tumor ≥ 4 cm in size, or grade 3, or with visceral pleura involvement), II or IIIA disease	ORR	June 26, 2018—November 2022
NCT04728724	PD-L1 expression	Sintilimab ± chemotherapy	II	100	III	MPR	January 21, 2021—December 31, 2027
NCT03425643	PD-L1 expression	Pembrolizumab + platinum doublet neoadjuvant chemotherapy	III	786	II, IIIA, and resectable IIIB (T3-4N2)	EFS, OS	April 24, 2018—June 29, 2026
NCT04865705	PD-L1 expression	Tilelizumab + Albumin Paclitaxel + Carboplatin/Cisplatin	II	33	III (T2N2, T3-4N1-2)	R0	November 10, 2020—June 10, 2022
NCT04326153	PD-L1 expression	Sintilimab + Albumin paclitaxel + Carboplatin	II	40	IIIA	DFS	December 15, 2019—December 2022
NCT04638582	PD-L1 expression	Pembrolizumab versus Pembrolizumab + platinum doublet chemotherapy	II	44	IA3, IB and IIA	ctDNA resolution	June 2021—March 2024
NCT04646837	PD-L1 expression	Durvalumab + Paclitaxel + Carboplatin	I/II	20	IB-IIIA	MPR	May 2021—March 2025
NCT04586465 (DYNAPET)	PD-L1 expression	Pembrolizumab + Pemetrexed + Gemcitabine + Cisplatin + Carboplatin	II	23	IIA—IIIB	MPR, dynamic SUV change, ORR, uptake rate constant (Ki)	October 10, 2020—June 30, 2022
NCT03800134 (AEGEAN)	PD-L1 expression	Durvalumab + platinum-based chemotherapy	III	800	IIA to select IIIB	pCR, EFS	December 6, 2018—April 30, 2024
NCT04379635	PD-L1 expression	Tislelizumab + Cisplatin/Carboplatin + Paclitaxel or Pemetrexed	III	380	II or IIIA	MPR, EFS	May 29, 2020—November 2025
NCT04338620	PD-L1 expression	Camrelizumab + albumin-bound Paclitaxel and Cisplatin	II	94	IIIA-IIIB	pCR	March 12, 2020—November 2021
NCT04840290	PD-L1 expression	Sintilimab + Platinum Doublet Chemotherapy	III	30	IIIA	ORR, R0 Resection Rate	June 1, 2019—June 1, 2023
NCT04699721	PD-L1 expression	Nivolumab + Paclitaxel + Carboplatin	I	40	IIIA and IIIB (T3N2)	Adverse effects, surgical complications, non-R0 surgical events	July 1, 2020—December 2027
NCT04459611 (neoSCORE)	PD-L1 expression	Sintilimab + Chemotherapy	II	60	IB-IIIA	MPR	July 1, 2020—July 1, 2023
NCT04025879	PD-L1 expression	Neoadjuvant Nivolumab + Platinum-based Doublet Chemo followed by Adjuvant Nivolumab	III	452	IIA-IIIB	EFS	September 16, 2019—September 30, 2024
NCT03732664	PD-L1 expression	Nivolumab + Pembrolizumab	I	40	IA3 (≥ 2 cm), II and IIIA	Safety and adverse effects	October 1, 2018—October 31, 2027
NCT04541251	PD-L1 expression	Camrelizumab + Nab-paclitaxel + Carboplatin	II	40	IB-IIIA	MPR	August 1, 2020—September 30, 2023
NCT04158440	PD-L1 expression	Toripalimab + Platinum doublet chemotherapy	III	406	IIIA	MPR, EFS	November 1, 2019—May 31, 2025
NCT04316364	PD-L1 expression	SHR-316 (anti-PD-L1 antibody) + Carboplatin + Paclitaxel	III	456	II, IIIA, IIIB	MPR, EFS	July 14, 2020—May 2026
NCT03916627	PD-L1 expression	Cemiplimab + Platinum doublet (NSCLC cohort)	II	94	Not specified	MPR, STN, MTE	July 23, 2019—August 7, 2027
NCT04865250 (iReP)	PD-L1 expression	Atezolizumab + Carboplatin/nab Paclitaxel	II	20	II, IIIA, IIIB	MPR	January 7, 2021—December 30, 2024
NCT04348292	PD-L1 expression	Durvalumab + Sirolimus	I	31	I, II, IIIa	Adverse events, pCR	March 10, 2021—December 25, 2025
NCT04560686	PD-L1 expression TGF-beta	Bintrafusp Alfa	II	23	I-IIIA disease and IIIB (T3N2 only, and N2 single station)	MPR	August 5, 2020—October 1, 2024
NCT04061590	PD-L1 expression	Pembrolizumab versus Pembrolizumab + Cisplatin/Pemetrexed	II	84	I-IIIA	Tumor-infiltrating immune cells (TIICs)	May 29, 2020—April 30, 2022
NCT04015778	PD-L1 expression	Nivolumab + Carboplatin/ nab-Paclitaxel	II	48	IB-IIIA	MPR	August 8, 2019—July 2024
NCT04108013	PD-L1 expression	SHR-1210 (PD-1 antibody) + Carboplatin + Paclitaxel-albumin	II	38	II-IIIA	MPR	October 8, 2019—July 31, 2021
NCT03623776	PD-L1 expression	JS001 (Toripalimab) + Pemetrexed/Carboplatin	II	80	IB (≥ 4 cm), II and IIIA	DFS	February 1, 2019—March 1, 2024

*Abbreviations: AE* adverse event, *ctDNA* circulating tumor DNA, *ORR* overall response rate, *pCR* pathological complete response, *MPR* major pathological response, *DFS* disease-free survival, *EFS* event-free survival, *PFS* progression free survival, *OS* overall survival, *MTE* major treatment effect, *STN* significant tumor necrosis, *TIICs* tumor-infiltrating immune cells. Blue highlights phase III studies

**Table 4:** Ongoing Neoadjuvant Clinical Trials Containing ICI with Either Other Immunotherapy or Targeted Therapy for Patients with resectable NSCLC

**ClinicalTrials.Gov Identifier**	**Biomarker(s)**	**Regimen**	**Phase**	**Sample Size**	**Stage**	**Primary Endpoint**	**Estimated or Actual Study Period [reference]**
NCT05472623 (Neo-Kan)	KRAS^G12C^ PD-L1 expression	Adagrasib BID prior to surgery versus Adagrasib BID and IV nivolumab every 2 weeks for 3 doses prior to surgery	II	42	IB-IIIA	pCR	November 20, 2022—November 20, 2025
NCT04941417	PD-L1 expression	PD-(L)1 inhibitor with chemotherapy	II	60	I (T ≥ 4 cm), IIA, IIB, IIIA, IIIB	MPR	November 24, 2020—June 30, 2023
NCT04379739	PD-L1 expression VEGF	Camrelizumab + platinum-based chemotherapy versus Camrelizumab + Apatinib	II	82	II-IIIA	MPR	July 26, 2020—December 30, 2026
NCT04875585 (INNWOP1)	PD-L1 expression VEGF	Pembrolizumab + Lenvatinib	II	33	IA3-IIIA	MPR	May 2021—December 2027
NCT04762030	PD-L1 expression	Durvalumab + Carboplatin + Nab-paclitaxel + Anlotinib	II	39	III	PFS	February 8, 2021—December 30, 2025
NCT04832854	PD-L1 expression	Atezolizumab + Tiragolumab with or without platinum-based chemotherapy	II	82	Stage II, IIIA, or select IIIB (T3N2 only)	Surgical delays, complications, surgical cancellations, adverse events, MPR	April 23, 2021—February 28, 2027
NCT04846634 (ALTER-L043)	PD-L1 expression VEGFR FGFR PDGFR c-kit	Penpulimab + chemotherapy versus Penpulimab + Anlotinib versus Penpulimab + chemotherapy + Anlotinib	II	90	IIB-IIIB (N2)	MPR	August 2021—February 2028
NCT04512430	PD-L1 expression VEGF	Atezolizumab + Bevacizumab + Carboplatin + Pemetrexed	II	26	IIIA	MPR	December 2, 2020—August 2026
NCT04506242	PD-L1 expression VEGF	Apatinib + neoadjuvant/adjuvant Camrelizumab	II	74	IIA-IIIB	MPR	September 15, 2020—December 30, 2025
NCT04123379	PD-L1 expression	Nivolumab + CCR2/5-inhibitor or anti-IL-8 (NSCLC cohort)	II	50	Not specified	MPR, STN	March 19, 2020—October 2024
NCT04040361 (EAST ENERGY)	PD-L1 expression VEGF	Pembrolizumab + Ramucirumab	II	24	IB-IIIA	MPR	November 30, 2019—November 30, 2025
NCT04205552 (NEOpredict)	PD-L1 expression LAG-3	Nivolumab + Relatlimab	II	60	I, II, IIIA	Feasibility	March 4, 2020 – June 2024
NCT03872661	PD-L1 expression VEGF	Sintilimab + Bevacizumab + Pemetrexed + Carboplatin	II	36	III	Resectibility rate	March 1, 2019—March 1, 2024
NCT03968419 (CANOPY-N)	PD-L1 expression IL-1β	Pembrolizumab ± Canakinumab or Pembrolizumab monotherapy	II	110	IB-IIIA	MPR	November 5, 2019—March 21, 2023
NCT04758949	PD-L1 expression IL-1ß	Nivolumab ± FL-101	II	90	IA3, IB, II, or IIIA	Adverse events	July 1, 2021—February 1, 2023
NCT04133337	PD-L1 expression VEGF	SHR-1210 (PD-1 Antibody) + Apatinib	I/II	20	IB-IIIA	MPR	November 1, 2019—June 2021

*Abbreviations: AE* adverse event, *SAE* severe adverse event, *pCR* pathological complete response, *MPR* major pathological response, *DFS* disease-free survival, *EFS* event-free survival, *PFS* progression free survival, *OS* overall survival, *STN* significant tumor necrosis

**Table 5:** Ongoing Neoadjuvant Clinical Trials Containing ICI with Radiation for Patients with NSCLC

**ClinicalTrials.Gov Identifier**	**Biomarker(s)**	**Regimen**	**Phase**	**Sample Size**	**Stage**	**Primary Endpoint**	**Estimated or Actual Study Period [reference]**
NCT03853187	PD-L1 expression	Durvalumab + Zr-89 labelled Durvalumab	II	20	I, II or IIIa	Safety and feasibility	September 19, 2019—April 1, 2022
NCT04933903	PD-L1 expression	Ipilimumab + Nivolumab + SBRT	II	25	IB-IIIB (T2-3N0, T1-T3N1-2)	MPR	July 2021—January 2023
NCT04271384	PD-L1 expression	SABR + Nivolumab	II	30	I	pCR	February 12, 2020—June 29, 2023
NCT02987998	PD-L1 expression	Cisplatin + Etoposide + Pembrolizumab with concurrent radiation	I	9	IIIA	Toxicity	May 18, 2017—January 24, 2024
NCT03871153	PD-L1 expression	Neoadjuvant chemotherapy + radiation + Durvalumab	II	25	III (N2)	PRR	August 2, 2019 – April 2022
NCT03217071	PD-L1 expression	Pembrolizumab + SRT	II	12	I-IIIA	Infiltrating CD3 + T cells/ μm2	October 4, 2017—February 28, 2022
NCT04465968 (DEEP_OCEAN)	PD-L1 expression	CRT + Durvalumab ± Surgery + Durvalumab	III	84	IIB, III	OS	September 1, 2020—August 31, 2030
NCT03965468 (CHESS)	PD-L1 expression	Durvalumab + Carboplatin/Paclitaxel Followed with either SBRT or surgery	II	47	IV	PFS	November 19, 2019—December 2021
NCT03237377	PD-L1 expression	Durvalumab + radiation	II	32	IIIA	Safety	December 12, 2017 – September 2017

*Abbreviations: CRT* concurrent chemoradiation therapy, *SBRT* stereotactic body radiation therapy, *pCR* pathological complete response, *MPR* major pathological response, *DFS* disease-free survival, *PFS* progression free survival, *OS* overall survival, *PRR* pathological response rate

### Neoadjuvant molecularly targeted therapies for oncogene-driven NSCLC

**Fig. 4:**
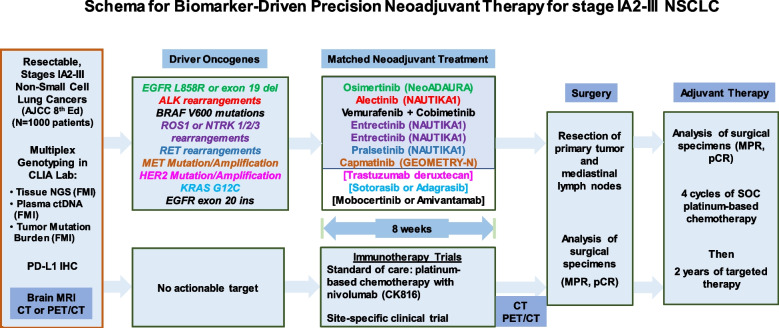
Schema for Biomarker-Driven Precision Neoadjuvant Therapy for stage IA2-III NSCLC. The LCMC4 (LEADER) screening trial is an umbrella trial that is designed to detect actionable oncogenic drivers by NGS in patients with resectable, early-stage NSCLC. Patients whose tumors harbor one of these oncogenic drivers are matched to one of the available, effective targeted therapies for patients with metastatic NSCLC. Those patients whose tumors do not harbor actionable driver oncogenes will receive standard of care or investigational neoadjuvant nivolumab and platinum-based chemotherapy

New generation targeted therapies have improved clinical outcomes in non-operatable metastatic NSCLC patients who were adherent to NCCN-recommended biomarker testing and first-line targeted therapy [77]. Asian patients with adenocarcinoma have higher prevalence of unique biologic features such as a higher incidence of oncogene-driven NSCLC (mainly EGFR-mutant and ALK-rearranged tumors), and the majority of Asian women with lung cancer are never-smokers. For patients with metastatic NSCLC, tumor genomic typing of NSCLC is important before starting the ICI therapy, not only because ICIs have low or inferior effect in EGFR-mutant or ALK-rearranged NSCLC, but also they have been associated with increased incidence and severity of interstitial lung disease and immune-mediated adverse effects (including pneumonitis, colitis and hepatitis) when they are in sequential or concurrent use with EGFR TKIs in patients with metastatic NSCLC [13, 78]. Given this concern, it is reasonable to perform genotyping of early-stage NSCLC tumors for targeted neoadjuvant therapy. Retrospective review of 40 patients with oncogene-driven NSCLC showed induction ICI therapy achieved a pCR rate of 12.5%, a MPR of 37.5% with a median EFS of 28.5 months [79]. NSCLC patients with sensitizing EGFR or ALK mutations were excluded in the CheckMate 816 trial [73]. Figure 4 summarizes the schema for an ongoing biomarker-driven precision neoadjuvant screening and matched targeted treatment trials for stage IA2-III NSCLC. The LCMC4 Evaluation of Actionable Drivers in EaRly Stage Lung Cancer (LEADER) screening trial (ClinicalTrials.gov Identifier: NCT04712877) builds on the Lung Cancer Mutation Consortium (LCMC)’s previous endeavors to now bring personalized neoadjuvant therapy to patients with early-stage lung cancers with actionable oncogenic drivers. This umbrella trial is designed to detect actionable oncogenic drivers in patients with suspected, resectable, early-stage NSCLC scheduled to undergo standard of care diagnostic biopsies to establish the diagnosis of lung cancer. Tumor samples are sent to next generation sequencing (NGS) for determining the presence of 11 oncogenic drivers (mutations in EGFR, BRAFV600E, MET exon 14, KRAS G12C, and HER2, rearrangements in ALK, RET, NTRK, and ROS1, and amplification of MET and HER2) in Clinical Laboratory Improvement Amendments (CLIA)-certified laboratories. Patients whose tumors harbor one of these oncogenic drivers are matched to one of the effective targeted therapies for patients with stage IV lung cancers. After 8 weeks of targeted therapy, patients will undergo surgical resection. Table 6 summarizes ongoing neoadjuvant clinical trials containing molecularly targeted therapeutics for patients with resectable NSCLC.

**Table 6:** Ongoing Neoadjuvant Clinical Trials Containing Molecularly Targeted Agents (MTAs) for Patients with NSCLC

**ClinicalTrials.Gov Identifier (Trial Name if available)**	**Biomarker(s)**	**Regimen**	**Phase**	**Sample Size**	**Stage**	**Primary Endpoint**	**Estimated or Actual Study Period [reference]**
NCT04181372	VEGFR FGFR PDGFR c-kit	Anlotinib hydrochloride + Platinum-based chemotherapy	II	50	II	Lymph node(N2) downstage rate	December 2019—August 1, 2022
NCT04351555 (NeoADAURA)	EGFR	Osimertinib ± Pemetrexed/Carboplatin or Pemetrexed/Cisplatin	III	328	II-IIIB (N2)	MPR	December 24, 2020—March 29, 2029
NCT04816838	EGFR	Osimertinib	Not stated	25	I-IIIA	ORR	March 2021- October 2025
NCT03349203	EGFR	Icotinib	II	60	IIIB or IV	ORR	October 1, 2018—December 30, 2023
NCT04926831	*MET* exon 14 skipping mutations	Capmatinib	II	38	IB-IIIA, N2 and selected IIIB (T3N2 or T4N2)	MPR	September 30, 2021—March 31, 2028
NCT04470076 (Neoafa)	EGFR	Afatinib + Pemetrexed, Gemcitabine + Cisplatin + Carboplatin	II	30	IIA-IIIB	MPR, ORR	July 10, 2020—July 30, 2023
NCT04455594 (ANSWER)	EGFR	Almonertinib	II	168	IIIA (N2)	ORR	October 2020—October 2025
NCT04302025	ALK RET ROS1 BRAF MEK1	Alectinib + chemotherapy versus Entrectinib + chemotherapy versus Vemurafenib + chemotherapy versus Cobimetinib + chemotherapy versus Pralsetinib + chemotherapy	II	60	IIA, IIB, IIIA, and select IIIB (T3N2)	MPR	November 6, 2020—August 15, 2028
NCT01059188	EGFR	Cetuximab + Cisplatin + Docetaxel + Radiotherapy	II	69	IIIB	PFS	May 3, 2010—September 2021
NCT03433469	EGFR	Osimertinib	II	27	I-IIIA	MPR	July 31, 2018—May 31, 2026
NCT02347839 (NEGOTIATE)	EGFR	Gefitinib	II	37	III (III A-bulky N2, III B)	Resectability rate	January 2016 – January 2022
NCT02820116	EGFR	Icotinib	II	67	IIA-IIIB	Complete resection rate	May 2016—April 2023
NCT04841811 (APPROACH)	EGFR	Almonertinib	II	156	III	ORR, EFS	August 15, 2021—August 15, 2024
NCT04648189	EGFR	Cetuximab	II	40	I-IIIA	CTCs	June 1, 2021—November 30, 2022
NCT01470716	EGFR	Erlotinib	II	26	II, IIIA	PFS	January 2012—September 2022
NCT03749213	EGFR	Icotinib	II	36	IIIA (N2)	ORR	December 1, 2018—December 30, 2024

*Abbreviations: CTC* circulating tumor cells, *ORR* overall response rate, *MPR* major pathological response, *EFS* event-free survival, *PFS* progression free survival, *OS* overall survival

### Pre-operative evaluation

**Fig. 5:**
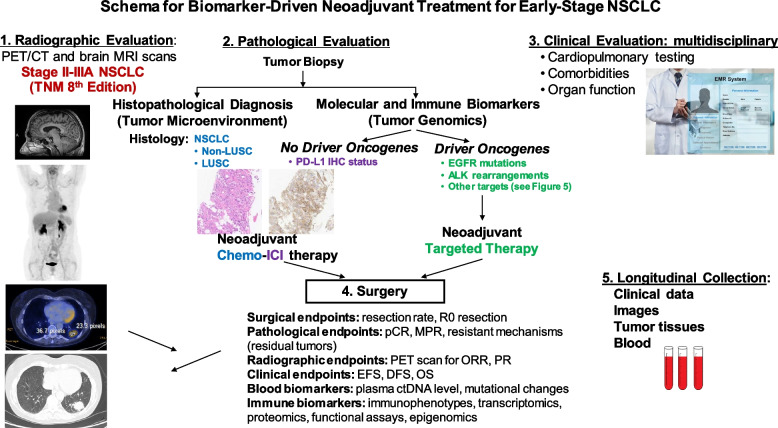
Schema for Biomarker-Driven Neoadjuvant Treatment for Early-Stage NSCLC. A multidisciplinary infrastructure is essential to implement biomarker-driven neoadjuvant treatment based on the histopathological and immunological evaluation and NGS-based molecular screening at the diagnosis of resectable NSCLC

Although lung resection is the cornerstone of curative treatment for lung cancer, there is an associated risk of mortality and morbidity with treatment. This is especially true for patients with impaired lung function, such as chronic obstructive pulmonary disease (COPD). Depending on the extent of lung resection, patients may have deterioration in their pulmonary function and may live with permanent changes that negatively impact their quality of life [80]. Therefore, comprehensive preoperative evaluation is an essential component to predict perioperative complications and long-term survival. Thoughtful consideration of surgical risks and benefits should be evaluated for each patient. The following section provides an overview of literature and a framework of current guidelines in the pre-operative evaluation process. Figure 5 summarizes the multidisciplinary evaluation for NSCLC patients for biomarker driven neoadjuvant therapy.

#### Radiographic evaluation for staging

Preoperative assessment begins with diagnostic imaging to localize the disease, visualize malignant involvement to adjacent structures, and determine the presence of metastasis. If an indeterminate nodule is seen on radiograph or chest CT, the first step is to compare the nodule with prior imaging. The most common staging system is the TNM staging system [7]. T refers to the size and extent of the main tumor (the primary tumor). N refers to the number of nearby lymph nodes that have cancer. M refers to whether the cancer has metastasized. Current recommendations state that further diagnostic testing is not warranted for solid nodules that have been stable for two years. On the other hand, if further work-up is indicated, combined ^18^F-FDG PET and CT has emerged as a valuable tool that is integral for all patients undergoing surgical evaluation [81, 82]. While CT scans can confirm the presence of disease, ^18^F-FDG PET used in conjunction provides insight into mediastinal lymph nodes, distant metastasis, and metabolic activity of the cancer. PET scans have been associated with higher accuracy, with both higher sensitivity and specificity than CT scanning alone [82]. Combined ^18^F-FDG PET/CT has been able to reduce the total number of thoracotomies and futile thoracotomies in NSCLC [83].

#### Tissue Biopsies

Histopathological evaluation of tumor biopsies is required not only for histologic diagnosis of lung cancer but also for molecular and immune biomarker analysis [84]. In the current era of precision lung cancer care, first-line systemic therapies for patients with metastatic NSCLC with good performance status are selected based on histology, and both molecular and immune biomarkers [85]. Furthermore, histopathological analysis of lymph nodes is essential for accurate pathological staging before surgery. Biopsy can be performed by bronchoscopy, mediastinoscopy, or needle biopsy [20]. Traditionally, mediastinoscopy has been the gold standard for pathological staging of mediastinal lymph nodes [86]. However, endobronchial ultrasound-guided transbronchial needle aspiration (EBUS-TBNA) has emerged as a well validated diagnostic tool demonstrated to have high accuracy. In patients with N2 disease, EBUS-TBNA had the ability for accurate nodal staging that could not be detected by either CT or PET/CT [87]. For patients undergoing diagnostic evaluation, EBUS-TBNA, compared to traditional bronchoscopy and transthoracic needle biopsy (TTNB), has been associated with lower complication rates, fewer required biopsies, and decreased need for repeat biopsies [88].

#### Cardiopulmonary Testing

Patients undergoing evaluation for resection often have underlying comorbidities. Cardiovascular diseases (CVDs) are the most frequently observed; this impacts up to 23% of patients with NSCLC and limiting survival [89, 90]. Currently, the American College of Chest Physicians Evidence-Based Clinical Practice Guidelines recommend cardiovascular evaluation for all patients undergoing preoperative evaluation for risk stratification, determination of operability, and prediction of perioperative complications [91].

Cardiac assessment begins with a thorough evaluation of the patient’s cardiac history and identification of risk factors that can predispose patients to adverse cardiac events. The thoracic revised cardiac risk index (ThRCRI) is a tool developed to evaluate cardiac risk specifically in thoracic patients undergoing lung resection and has shown to have good discrimination [92]. The ThRCRI is a weighted score of four risk factors: renal status (creatinine > 2 mg/dL), ischemic heart disease, cerebrovascular disease, and pneumonectomy [93]. Based on the cumulative score, patients can either proceed with surgical resection or undergo further evaluation to assess cardiac function. Any patient who has a ThRCRI score of ≥ 2, cardiac conditions that require monitoring, a newly suspected cardiac condition, and/or or an inability to climb 2 flight of stairs require evaluation by a cardiologist and optimization of cardiac function prior to surgery.

In addition to a comprehensive cardiac evaluation, pulmonary function tests are a non-invasive approach to inform the extent of resection that is feasible and evaluate long-term survival [94]. The two primary metrics used to determine pulmonary function include FEV_1_ and D_LCO_, as well as their respective predicted postoperative (PPO) values. PPO is calculated by estimating the proportion of the lung being resected and subtracting the value from baseline FEV_1_ and D_LCO_. In patients with either PPO FEV_1_ or PPO D_LCO_ > 60%, they are considered low risk and can proceed to surgery without requiring further testing. On the other hand, if either the PPO FEV_1_ or PPO D_LCO_ < 60%, further evaluation of baseline cardiopulmonary function is recommended. Ensuring adequate cardiopulmonary reserves is critical as low FEV_1_, PPO FEV_1_, D_LCO_ and PPO D_LCO_ values have been associated with increased risk of morbidity and mortality [95, 96]. Specifically, PPO FEV_1_ and D_LCO_ < 40% has been used as a cut-off value associated with an independent predictor of mortality with surgery [97].

In patients with diminished pulmonary function tests, the current recommendation is to pursue exercise testing and evaluate maximal oxygen consumption (VO_2max_). Determining baseline cardiopulmonary reserve is necessary, as patients with lung cancer often have low baseline exercise capacity due to underlying atherosclerotic disease and exposure to cigarette smoking. Decreased cardiopulmonary function at baseline is concerning, as lung function often decreases after resection [98]. This is compounded by the fact that patients are exposed to cardiotoxic treatments, causing radiation-induced cardiovascular disease and cardiac dysfunction associated with commonly used platinum-based antineoplastic agents [99]. While exercise capacity can be described subjectively, Cardiopulmonary Exercise Testing (CPET) is valuable as an objective measurement that can quantify cardiopulmonary function, subsequently allowing stratification of patients based on distinct cut-off values. Furthermore, CPET elucidates the effectiveness of the patient’s compensatory mechanisms to maintain adequate circulation and oxygenation with increasing physiological stress. If CPET is unavailable, alternative validated methods that have been investigated include the 6-min walk test (6MWT), shuttle walk test (SWT), and stair-climbing test (SCT) [100].

In CPET, patients exercise with either a treadmill or cycle ergometry at incremental workloads while maximal oxygen consumption (VO_2max_), maximal carbon dioxide consumption (V_CO2_), minute ventilation (V_e_), oxygen saturation, heart rate, and blood pressure are measured or calculated [101]. Among the variables measured, VO_2max_ has shown to be the greatest predictor of postoperative complications and used to determine surgical risk. In patients unable to achieve VO_2max_, peak oxygen consumption (VO_2 peak_) can be used as a surrogate. A patient with VO_2max_ > 20 mL/kg/min is deemed low risk, 10–20 mL/kg/min is moderate risk, and < 10 mL/kg/min is high risk [100, 102]. In patients with VO_2max_ < 10 mL/kg/min, alternative therapies to surgery and/or minimal resection should be considered due to the high risk of mortality associated with major resection [103].

##### Other factors affecting surgery

Lung cancer survival rates vary by geographic location, histologic subtype, stage at diagnosis, and treatment [2]. Many additional factors, such as the COVID pandemic, socioeconomic status, timely access to health care, area of residence, and immigration status can also contribute to differential incidence and mortality by race [104]. Stage III NSCLC is a heterogenous group of disease that has significant variations in the selection of treatment modality. Currently, only 21% of patients with stage III NSCLC undergo surgery, whereas most (61%) are treated with chemotherapy and/or radiation [10]. In a recent report, surgery is substantially lower among Black patients with NSCLC with 49% for stages I-II and 16% for stage III compared to 55% and 22% for White patients, respectively [10]. This treatment disparity is also exacerbated by the fact that Black patients continue to be less likely to be diagnosed with stage I disease than White patients for most cancers. As the benefit of neoadjuvant systemic therapy is most prominent in patients with stage III NSCLC, this practice disparity needs to be considered.

### Evaluation of neoadjuvant therapy

Although overall survival remains the gold standard of endpoints for neoadjuvant clinical trials, it will take a decade to mature. Several surrogate endpoints that have been used for evaluating neoadjuvant chemotherapy are being explored in the neoadjuvant ICI trials.

#### Surgical endpoints

The surgical endpoints for neoadjuvant therapy are feasibility of surgery, complete surgery, and minimally invasive surgery. Currently, more than one-half (55%) of patients with stage I or II NSCLC undergo surgery with either sublobar resection (partial removal of a lobe of the lung), sleeve resection (removal of the tumor and a portion of the affected airways), lobectomy (entire removal of an affected lobe), or pneumonectomy (removal of one lung). There are many factors to consider when discussing or planning for neoadjuvant therapy in lung cancer patients that should be evaluated. First is feasibility of surgery. We must consider patient factors which include, but are not limited to, comorbid conditions, pulmonary function, suitability for surgery, and overall functional status. Second, are tumor characteristics including location of the tumor. Is the tumor peripheral or central; will it require segmentectomy, lobectomy, bilobectomy, or pneumonectomy? In some cases, en bloc chest wall resection and reconstruction may need to be considered in order to achieve an R0 resection. In addition to these endpoints, there are other factors to consider, such as invasiveness of surgery. It is well known that there are several benefits and advantages that are attributed to minimally invasive approaches over thoracotomy which include video-assisted thoracic surgery (VATS) and robot-assisted thoracic surgery (RATS). These benefits include faster recovery time, shorter hospital stay, decreased pain, smaller incisions, and fewer complications.

Neoadjuvant therapy may lead to certain surgical challenges which have been reported such as the presence of significant inflammation and fibrosis which have ultimately led to longer operative times and more conversions from a minimally invasive to an open operation [105]. It is notable however, that this observation did not translate to a substantially higher rate of postoperative complications than for other studies with neoadjuvant chemotherapy [106]. In addition, immune-mediated side-effects, such as pneumonitis, can delay or prevent surgery, and can also complicate the postoperative period [66].

#### Clinical endpoints

The gold standard of neoadjuvant therapy is OS. EFS or DFS is a surrogate endpoint of OS for neoadjuvant chemotherapy, which takes a long time to mature. Several clinical trials demonstrated that pCR to neoadjuvant chemotherapy has been associated with improved survival in retrospective studies [107, 108]. Based on these data, the US FDA has accepted pCR as an endpoint for the approval of new drugs in the neoadjuvant study. This strategy can markedly shorten the time and costs needed in the new drug development in the neoadjuvant setting. The role of EFS or DFS as the clinical endpoints for cure and OS remains to be defined in precision neoadjuvant targeted and immunotherapy for resected, early-stage NSCLC.

#### Pathological endpoint

Major pathologic response (MPR) was defined as a residual viable tumor of less than or equal to 10% [107]. Both pCR and MPR were established from the neoadjuvant chemotherapy trials and serve as surrogate endpoints for OS for patients with resectable tumors after neoadjuvant chemotherapy. Their roles in neoadjuvant immunotherapy need to be further defined [109].

#### Radiographic evaluation

**Fig. 6:**
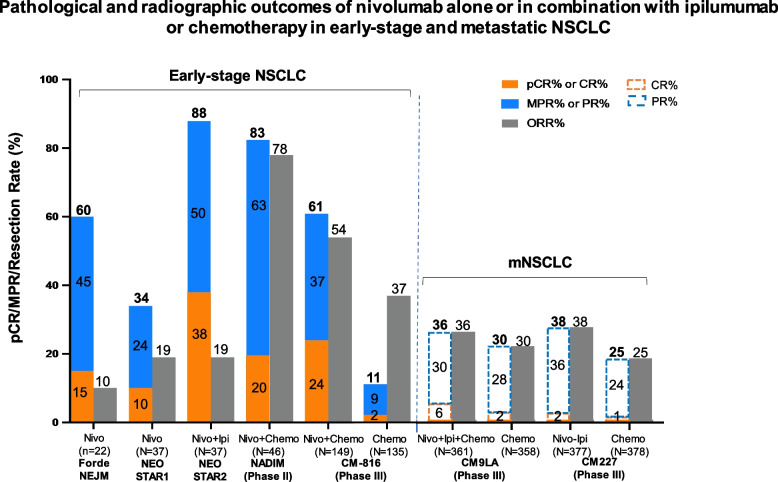
Pathological and radiographic outcomes of nivolumab alone or in combination with ipilumumab or chemotherapy in early-stage and metastatic NSCLC. The pathologic complete response (pCR, solid bar in orange) and major pathologic response (MPR, solid bar in blue) rate (%) by histopathological evaluation were shown in resectable, early-stage NSCLC. No pathological stage is available for patients with unresectable NSCLC. The overall response rate (ORR) is the radiographic evaluation of tumor response by RECIST, which is higher in early-stage NSCLC compared to mNSCLC. Per RECIST V1.1, CR (dashed bar in orange) is defined as disappearance of all targeted lesions, and any pathological lymph nodes (whether target or non-target) must have reduction in short axis to < 10 mm. Partial response (PR, dashed bar in blue) is defined as at least a 30% decrease in the sum of diameters of target lesions, taking as reference the baseline sum diameters. This PR criteria correlate with at least a 50% decrease in tumor volume

^18^F-FDG PET/CT scan has been used in initial staging at diagnosis of early-stage NSCLC [81, 82]. In radiographic evaluation for patients with advanced or metastatic NSCLC, ORR is the sum of CR and PR per Response Evaluation Criteria in Solid Tumors (RECIST) [110, 111]. However, in neoadjuvant NSCLC studies, radiographic response by conventional CT or PET/CT scans seems to underestimate the tumor response by pathological evaluation (Fig. **6**). This was first noticed in neoadjuvant nivolumab study, -3 patients achieved pCR, but none were evident by CT imaging; one patient even had apparent tumor growth radiologically. This phenomenon was called “pseudo progression” due to immune cell infiltrates in patients with advanced solid tumors receiving ICI therapy. In the phase III study of CM-816, ORR was higher than the sum of pCR and MPR [107, 108]. Improved radiographic evaluation for predicting pathological response to neoadjuvant treatment is needed. Furthermore, the dynamic changes in cancer metabolism to targeted therapy, immunotherapy, or chemoimmunotherapy might be different. Ongoing studies are evaluating better tracers [109] for differentiating tumor cells from immune cells or sensitive scanner [112] to improve the in vivo assessment of cancer metabolism to precision neoadjuvant therapy in NSCLC.

### Correlative studies

#### Tissue biomarker endpoints

Although genotyping of plasma circulating tumor DNA (ctDNA) has received US FDA approval and has been increasingly used to complement tissue-based genomic assays in precision oncology, pathohistological assessment of tumor specimens remains the gold standard for diagnosis of lung cancer. High TMB and high interferon-gamma-related gene expression signature score (IFN-γ score) were associated with pathologic response and low risk of relapse [113]. A meta-analysis included 10 studies involving 461 NSCLC patients. Compared with PD-L1 expression < 1%, PD-L1 expression ≥ 1% was associated with a higher rate of MPR and pCR. High TMB was also associated with MPR and pCR. Similar findings were observed in subgroup analyses, regardless of PD-1/PD-L1 inhibitor monotherapy or their combination with chemotherapy. Notably, 50% as the cutoff value for PD-L1 expression demonstrated better prediction efficacy for MPR than that of 1% [114]. A recent whole exosome analysis of 14 NSCLC patients (3 adenocarcinoma and 11 squamous cell carcinoma) who achieved MPR with chemoimmunotherapy, the detection of homologous recombination deficiency (HRD) was associated with enhanced response to neoadjuvant immunotherapy [115]. Further studies are needed to validate the results in prospective neoadjuvant immunotherapy studies.

#### Blood biomarker endpoints

Effective neoadjuvant therapy can result in insufficient viable tumor cells left in surgical specimens for biomarker testing and selection for adjuvant therapy. Increasingly, liquid biopsy and plasma ctDNA assays have been used as a minimally invasive way to study tumor biology and monitor dynamic changes of molecular and immune biomarkers during cancer treatment [116]. First, undetectable ctDNA has been associated with elimination of micrometastasis and clinical remission. In the CK-816 study, ctDNA clearance was higher in the 24 patients that received nivolumab-chemotherapy than the 19 patients received chemotherapy alone (56% versus 35%). Those patients with ctDNA clearance had longer EFS (HR 0.6) and higher pCR rate (HR 0.13) compared to those without ctDNA clearance. A recent prospective study enrolled 261 patients with stages I to III NSCLC who underwent surgical resection and evaluated the prognostic value of ctDNA-MRD detection during the surveillance of NSCLC. Those patients who maintained longitudinal undetectable MRD for more than 18 months could be classified as the potentially cured population. However, most current ctDNA analysis methods lack adequate sensitivity for residual disease detection during and after completion of treatment in cancer patients, along with high cost [117]. Patients with early-stage NSCLC had lower plasma ctDNA compared to those with metastatic NSCLC for tumor genotyping. Furthermore, some patients with ctDNA clearance still had cancer recurrence. Thus, highly sensitive blood tests are needed to detect low quantity of systemic micrometastasis in the blood after surgery. Extracellular vesicles such as exosomes play a role in cancer progression by transferring bioactive molecules to non-tumorigenic and tumorigenic recipient cells [118–120]. Tumor-derived exosomes isolated from the plasma of patients contain high quality nucleic acids (DNA, RNA, and miRNA), which is suitable for tumor genomic typing [121]. Thus, detecting tumor-derived exosomes in patients’ plasma after surgery might serve as a surrogate for residual micrometastasis. Furthermore, integrins mediate the organ-specific metastases of circulating tumor cells and exosomes [118, 122, 123]. Ongoing studies are evaluating if ctDNA detection may inform a personalized approach to duration of PD-(L)1 treatment in patients with long-term response. For those patients with undetectable ctDNA, they may be able to safely discontinue treatment. Additional work will be needed to determine the optimal timepoint (e.g., 12–24 months after initiating treatment) at which to query ctDNA to guide such a decision.

**Fig. 7:**
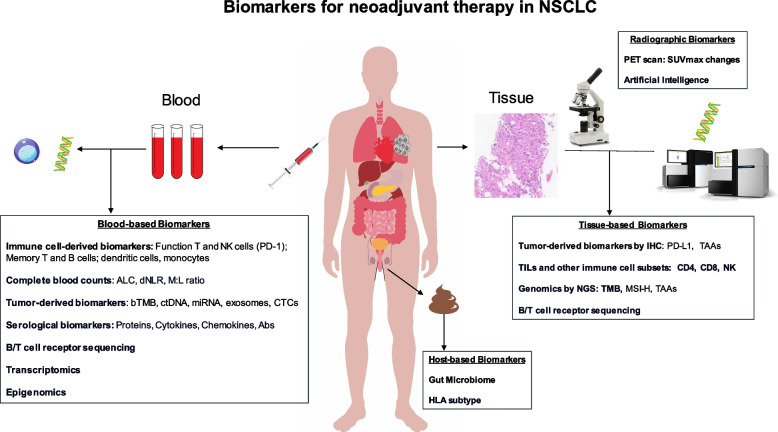
Biomarkers for neoadjuvant therapy in NSCLC. Schema illustrates the emerging biomarkers of tumor tissue, blood, host, and radiology for neoadjuvant therapy in NSCLC

Additionally, lymphocyte counts and immunophenotyping of T-cell and B-cell have been used to assess the immune status and are prognostic biomarkers. Low levels of lymphocytes in the blood could indicate an increased risk for death [124]. In NSCLC patients who had MPR, lower baseline levels of T cells and NK cells, but after neoadjuvant therapy, these patients experienced an expansion of NK cells and granulocytes and increased abundance of DC and B cells in lymph nodes, as well as decreased abundance of monocytes, suggesting that immunotherapy plays a key role in preoperative activation of tumor-specific immune killing [125]. Several studies have shown that pre-treatment low ALCs (< 600–1200 cells/µL) were associated with decreased PFS and OS to ICI in NSCLC patients [126–129]. Dynamic changes of ALCs after ICI treatment were also associated with clinical response. Post-ICI treatment low ALCs (< 700–900 cells/µL) were associated with decreased PFS and OS in patients with advanced solid cancer types including NSCLC [127, 130]. Immunophenotypic analysis of circulating immune cells revealed increases in circulating proliferating CD4 + and CD8 + T cells at 2 weeks after durvalumab treatment [131]. Assessing the function of peripheral T-cell subclones, particularly the T-cell receptor (TCR) clonality and activity to clonal neoantigens, have also been explored as a predictive biomarker for response to ICI [131, 132]. Furthermore, high derived neutrophil-to-lymphocyte ratio (dNLR) was associated with poor prognosis in patients with advanced NSCLC treated with durvalumab [133]. Figure **7** summarizes the emerging biomarkers of tumor tissue, blood, host, and radiology for neoadjuvant therapy in NSCLC. Further studies are needed to evaluate the role of these blood biomarkers in guiding neoadjuvant/adjuvant therapy.

## Summary and perspectives

Neoadjuvant therapies hold great promise to improve surgical and pathological outcomes, reduce local and systemic recurrences, guide postoperative therapy, and improve cure rates in patients with resectable NSCLC. The biomarker-driven neoadjuvant treatment extends the precision oncology from advanced stage to early-stage NSCLC. This is particularly important for those stage IIIA patients with N2 disease who are mostly (61%) treated with chemotherapy and/or radiation [10]. Table **7** summarizes the advantages and disadvantages of neoadjuvant chemoimmunotherapy in patients with resectable NSCLC. The preoperative evaluation and timely biomarker testing, in the era of precision neoadjuvant therapy for early-stage NSCLC, requires a paradigm shift. This paradigm shift will require the collaboration of thoracic surgeons, medical oncologists, interventional pulmonologists, radiologists, and pathologists. In addition, the emerging neoadjuvant chemoimmunotherapy has brought several new radiographic, pathological, and clinical endpoints that would need further evaluation. Further research studies are required to compare neoadjuvant and adjuvant treatment, optimize biomarker guided treatment selection, and define the treatment duration to improve the survival of early-stage NSCLC.

**Table 7:** Advantages and disadvantages of neoadjuvant immunotherapy

Advantages	Disadvantages
⦁ Completion of more systemic therapy before surgery than after surgery ⦁ Early elimination of micrometastasis and prevention of tumor recurrence ⦁ Increase in R0 resection rate ⦁ Downstage of tumor (i.e., Decrease in the TNM stage) ⦁ Assessment of the in vivo effect of systemic therapy (i.e., sensitivity or resistance) by radiographic and pathological evaluations when the tumor is intact ⦁ Understanding of the resistance mechanism(s) for residual tumor and select potential adjuvant therapy ⦁ Immune modulation leading to antigen priming and counteracting immunosuppressive effect of surgery ⦁ Shorter time of ICI treatment compared to adjuvant ICI therapy ⦁ More time for physical and psychological preparation for surgery (i.e., control comorbidities) and smoking cessation	⦁ Requirement of a timely multidisciplinary collaboration of patient care ⦁ Risk of tumor progression during neoadjuvant treatment ⦁ Toxicities, including bone marrow suppression and immune mediated adverse effects, might delay to curative surgery ⦁ Increased technical difficulty and risks with the operation due to various degrees of fibrosis at the trachea and pulmonary vasculature ⦁ Prognostic significance of MPR not well established ⦁ Lack of validated predictive biomarkers to select the patients for neoadjuvant ICIs ⦁ Lack of validated predictive biomarker to predict the success of surgery (i.e., hyper-progression induced by immunotherapy)

*Abbreviations: TNM* tumor, node, metastasis, *ICI* immune checkpoint inhibitor, *MPR* major pathological response

## Data Availability

Data sharing not applicable to this article as no data-sets were generated or analyzed during the current study.

## Abbreviations

6MWT: 6-Minute walk test
AACR: American Association for Cancer Research
ACTH: Adrenocorticotropic hormone
AJCC: American Joint Committee on Cancer
ALCs: Absolute lymphocyte counts
ALK: Anaplastic lymphoma kinase
ANC: Absolute neutrophil counts
ASCO: American Society of Clinical Oncology
BRAF: V-raf murine sarcoma viral oncogene homolog B1
CI: Confidence interval
CLIA: The Clinical Laboratory Improvement Amendments
CPET: Cardiopulmonary Exercise Testing
ctDNA: Circulating tumor DNA
CTCAE: Common Terminology Criteria for Adverse Events
CTLA: Cytotoxic T-lymphocyte–associated antigen
CVD: Cardiovascular disease
DC: Dendritic cell
DFS: Disease free survival
D_LCO_: Carbon Monoxide Diffusing Capacity
DNA: Deoxyribonucleic acid
dNLR: Derived neutrophil-to-lymphocyte ratio
EBUS-TBNA: Endobronchial ultrasound-guided transbronchial needle aspiration
EFS: Event free survival
EGFR: Epidermal growth factor receptor
ERBBR2: Erb-B2 Receptor Tyrosine Kinase 2
FDG PET: Fluorodeoxyglucose-positron emission tomography
FDA: Food and drug administration
FEV1: Forced expiratory volume in the first second
GEP: Gene expression profile
HER2: Human epidermal growth factor receptor 2
HLA-DR: Human leukocyte antigen – DR
HPA: Hypothalamic–pituitary–adrenal axis
HR: Hazard ratio
ICIs: Immune checkpoint inhibitors
IFN-γ: Interferon γ
IHC: Immunohistochemistry
kg: Kilogram
KRAS: Kirsten rat sarcoma virus
LCMC4: Lung cancer mutation consortium 4
LEADER: LCMC4 Evaluation of Actionable Drivers in EaRly Stage Lung Cancer
LUAD: Lung adenocarcinoma
LUSC: Lung squamous cell carcinoma
MET: Mesenchymal‐to‐epithelial transition
miRNA: MicroRNA
mL: Milliliter
mNSCLC: Metastatic non-small cell lung cancer
MPR: Major pathologic response
MRD: Minimal residual disease
N: Number
NCCN: National Comprehensive Cancer Network
NCI: National Cancer Institute
NGS: Next generation sequencing
NK: Natural killer
NKCA: Natural killer cell activity
NTRK: Neurotrophic receptor tyrosine kinase
NOS: Not otherwise specified
ORR: Overall response rate
NSCLC: Non-small cell lung cancer
OS: Overall survival
PD-L1: Programmed death-ligand 1
PET/CT: Positron emission tomography-computed tomography
PD: Progression disease
PFS: Progression free survival
PR: Partial response
PPO: Predicted postoperative
RATS: Robot-assisted thoracic surgery
RET: Rearranged during transfection
RECIST: Response Evaluation Criteria in Solid Tumors
RFS: Recurrence-free survival
RNA: Ribonucleic acid
ROS1: ROS proto-oncogene 1, receptor tyrosine kinase
SBRT: Stereotactic body radiotherapy
SCT: Stair-climbing test
SD: Stable disease
SD: Standard deviation
SWT: Shuttle walk test
TCR: T-cell receptor
Th1: T helper cell type 1
Th2: T helper cell type 1
ThRCRI: Thoracic Revised Cardiac Risk Index
TILs: Tumor infiltrating lymphocytes
TKIs: Tyrosine kinase inhibitors
TMB: Tumor mutation burden
TME: Tumor micro environment
VATS: Video-assisted thoracoscopic surgery
VEGF: Vascular endothelial growth factor
VO_2_max: Maximal oxygen consumption
WCLC: The World Conference on Lung Cancer
